# 951 Equipping People Who Are Unhoused with Fire, Burn, and Cold Injury Prevention Education

**DOI:** 10.1093/jbcr/iraf019.482

**Published:** 2025-04-01

**Authors:** Caitlin Orton, Tony Machacha, Carly Marincasiu, Maiya Pacleb, Megan Moore, Barclay Stewart

**Affiliations:** University of Washington; King County Regional Homelessness Authority; Harborview Medical Center, University of Washington; University of Washington Medicine Regional Burn Center; University of Washington; University of Washington

## Abstract

**Introduction:**

Almost a quarter of people receiving care for major burn injuries in urban burn centers across North America are unhoused at the time of injury. Prevention of these injuries, especially for populations living unhoused, requires the delivery of passive and active fire and cold weather projections along with education. For populations with low health literacy, it is extremely important that prevention education is written in plain language, contextualized, and consumer tested to increase a material’s acceptability, understandability, and actionability. We aimed to consumer test newly developed fire, burn, and cold injury prevention education materials with people experiencing homelessness (PEH) and identify PEH preferred prevention strategies to address and mitigate fire and cold weather risks and hazards.

**Methods:**

40 cognitive interviews with PEH were conducted. The Patient Education Materials Assessment Tool (PEMAT-P) and the Model System Knowledge Translation Center (MSKTC) consumer testing toolkit were used to evaluate the understandability and actionability of the six newly developed education materials. Transcripts were analyzed using a harm reduction lens and a combination of deductive and inductive thematic coding.

**Results:**

Participants provided feedback on the understandability and actionability of newly developed educational materials. Feedback themes were categorized into the domains of 1) engage and relate – being relatable to the diverse experiences and literacy levels of PEH, 2) reduce harm – focus on mitigating rather than eliminating hazards, 3) use context-specific design – reflect the lived experiences of PEH and their environments in the materials, 4) empower – incorporate prevention guidance, tips and tricks from PEH in addition to conventional prevention strategies, and 5) prioritize both equipment and education – combine education with dissemination of safety equipment (e.g., fire blankets, cookstove adaptations, clothing).

**Conclusions:**

The process of consumer testing with PEH generated acceptable and actionable prevention education materials and identified specific fire, burn and cold injury prevention and mitigation strategies for people living unhoused. Additionally, consumer testing increases collective support and trust from PEH and the organizations that work with them.

**Applicability of Research to Practice:**

By developing and implementing targeted burn and cold injury prevention education in conjunction with PEH preferred prevention and mitigation strategies, public health initiatives will be strengthened and may be more effective.

**Funding for the Study:**

This project was funded by a Population Health Initiative Grant and the David and Nancy Auth-Washington Research Foundation Endowed Chair for Restorative Burn Surgery.

